# The effect of marital status on cervical cancer related prognosis: a propensity score matching study

**DOI:** 10.1038/s41598-025-19122-3

**Published:** 2025-10-08

**Authors:** Kaige Pei, Mingrong Xi

**Affiliations:** 1 Department of Gynecology and Obstetrics, West China Second University Hospital, Sichuan University, No. 20, Section 3, Renmin South Road, Wuhou District, Chengdu, Sichuan Province China; 2 Key Laboratory of Birth Defects and Related Diseases of Women and Children, Sichuan University, Chengdu, Sichuan Province China

**Keywords:** Cervical cancer, Marital status, Survival analysis, Propensity score matching, Cox regression model, Cancer, Diseases, Oncology, Risk factors

## Abstract

Cervical cancer is the fourth most common malignancy in women worldwide and the fourth leading cause of cancer death in women. Although an association between marital status and prognosis has been observed in a variety of malignancies, this link has not been fully elucidated in the field of cervical cancer. A total of 30,853 patients were enrolled in this study, of whom 14,286 (46.3%) were married and 16,567 (53.7%) were unmarried. The median follow-up time was 76 months. Before propensity score matching (PSM), cancer-specific survival (CSS) (HR = 1.55, 95% CI 1.49–1.61, *p* < 0.001) and overall survival (OS) (HR = 1.66, 95% CI 1.61–1.73, *p* < 0.001) in unmarried patients were significantly lower than those in married patients. After 1:1 PSM matching, there were 10,771 patients in each of the two groups, and baseline features were balanced (*p* > 0.05 for all variables). Even after PSM, CSS (HR = 1.07, 95% CI 1.02–1.13, *p* = 0.003) and OS (HR = 1.13, 95% CI 1.08–1.18, *p* < 0.001) of unmarried patients were significantly lower than those of married patients. Multivariate Cox regression analysis confirmed that unmarried status was an independent risk factor for CSS (HR = 1.12, 95% CI 1.07–1.17, *p* < 0.001) and OS (HR = 1.19, 95% CI 1.14–1.24, *p* < 0.001). Based on the large sample size analysis, this study revealed the association between unmarried status and poor prognosis of cervical cancer, and confirmed that marital status is an independent factor affecting the prognosis of cervical cancer. Nevertheless, more prospective studies and in-depth mechanistic studies are needed to validate our findings and explore the biological basis behind them.

**Supplementary Information:**

The online version contains supplementary material available at 10.1038/s41598-025-19122-3.

## Introduction

Cervical cancer is the fourth most common malignancy among women worldwide and occupies the same position among the causes of cancer death in women^1^. In 2020, there will be approximately 600,000 new cases of cervical cancer and 340,000 deaths due to cervical cancer worldwide^2^. Squamous cell carcinoma (SCC), adenocarcinoma (AC), and adenosquamous carcinoma (ASC) are the three most common histological types of cervical cancer. SCC accounts for about 80% of all cervical cancers. Regardless of the histological type and HPV infection status of patients with cervical cancer, the current mainstream treatment options are surgery, radiation, chemotherapy, or a combination of the above treatments^3^. Despite the introduction of cervical cancer vaccine and cervical cancer screening for a long time, there are still more new cases of cervical cancer around the world, and even the proportion of young cervical cancer patients in some countries is increasing^1^, which indicates that cervical cancer is still a serious threat to women’s health worldwide.

Current studies have shown that the prognosis of cervical cancer is related to a variety of factors, mainly the histopathological features of the tumor, such as the size of the primary tumor, lymph node metastasis, lymphatic-vascular space infiltration, staging, and histological grade^4–12^. Secondly, some laboratory indicators and their derivatives are also related to the prognosis of cervical cancer^13–22^. With the rise of bio-psychosocial medical models, psychological and social factors have become increasingly important in cancer research^23^. It is worth noting that marital status has been shown to be associated with the prognosis of various cancers^24^, such as prostate cancer^25,26^, early liver cancer^27^, breast cancer^28^, pancreatic ductal carcinoma^29^, glioblastoma^30^, and non-small cell lung cancer^31^. Although some previous studies have initially explored the impact of marital status on the prognosis of cervical cancer^32–34^, due to the different control methods of confounding factors, there may be a certain degree of bias. Therefore, there is a lack of a comprehensive study to delve into the link between marital status and cervical cancer prognosis, highlighting the importance of our study. Identifying the link between marital status and cervical cancer prognosis is critical, not only to help clinicians take marital status into account when making treatment plans in order to achieve better treatment outcomes, but also to reveal potential biological mechanisms affecting prognosis and provide directions for exploring new treatment strategies.

In this study, we used data from the Surveillance, Epidemiology, and End Results (SEER) database, the largest cancer database in the world, to assess the difference in outcomes between married and unmarried cervical cancer patients. Through this study, we expect to provide new insights into the field of cervical cancer treatment and lay the foundation for future research directions.

## Results

### Patient baseline characteristics

**Fig. 1:**
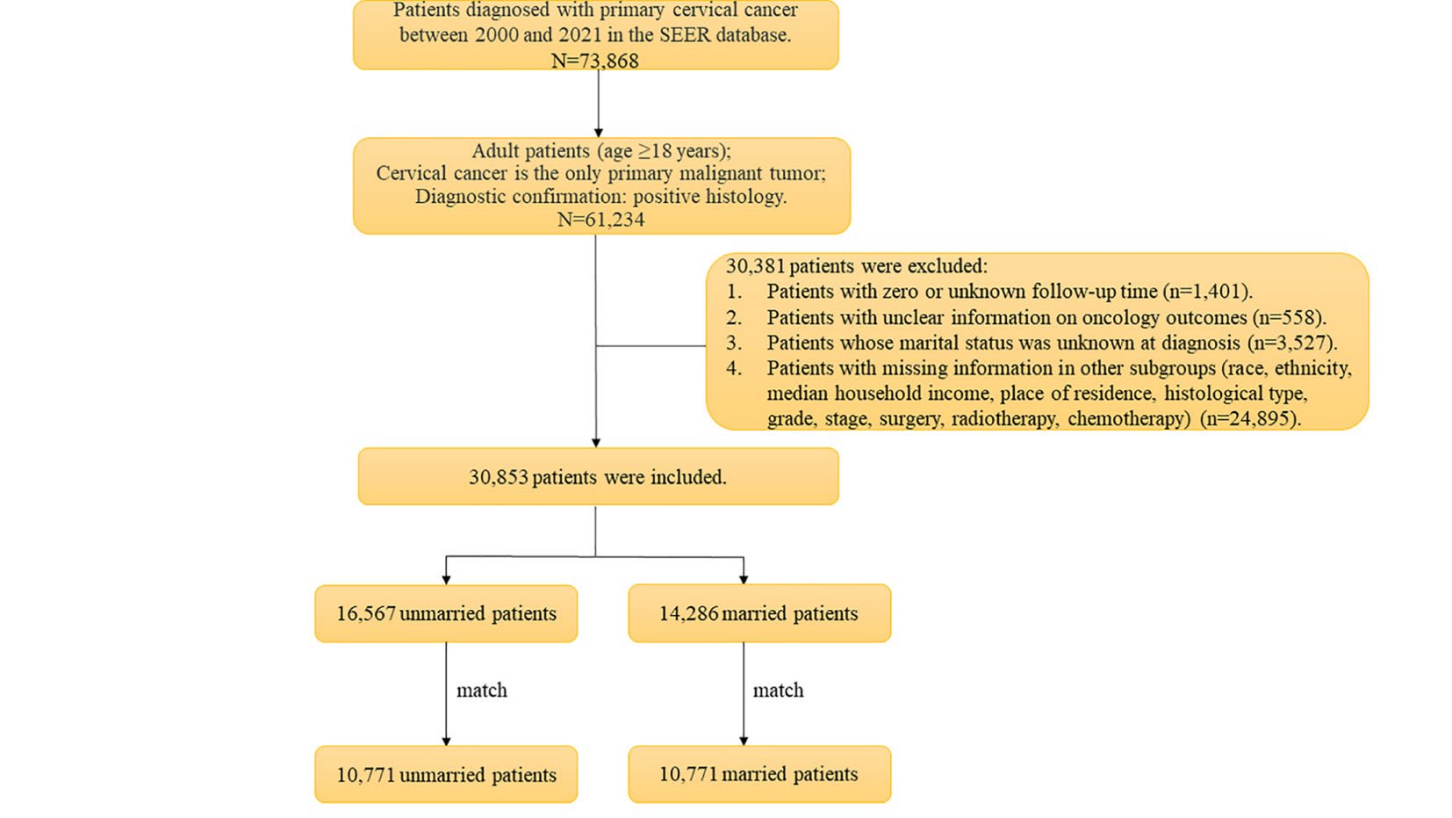
Patient screening flowchart.

The patient selection process is shown in Fig. 1. The baseline characteristics of the included patients are shown in Table 1. A total of 30,853 patients were included, with a median follow-up of 76 months. Among them, 16,567 (53.7%) were unmarried and 14,286 (46.3%) were married. A total of 15,154 patients (49.1%) were diagnosed from 2000 to 2008 and 15,699 (50.9%) were diagnosed from 2009 to 2017. There were 13,458 patients (43.6%) aged < 45 years, 10,886 patients (35.3%) aged 45–60 years, and 6,509 patients (21.1%) aged > 60 years. In terms of demographics, most patients were white (76.6%), non-Hispanic (75.8%), had a median household income of less than $75,000 (54.6%), and lived in an urban area (88.2%). In terms of tumor characteristics, squamous cell carcinoma, adenocarcinoma and other histological types accounted for 66.9%, 22.5% and 10.6% of the total population, respectively. There were 4332 (14.0%) well-differentiated patients, 13,055 (42.3%) moderately differentiated patients, and 13,466 (43.7%) poorly differentiated or undifferentiated patients. For cancer stage, 14,347 (46.5%) patients were local, 12,326 (40.0%) patients were regional, and 4180 (13.5%) patients were distant stages. In terms of treatment, 18,700 patients (60.6%) received surgery, 18,443 patients (59.8%) received radiotherapy, and 15,537 patients (50.4%) received chemotherapy.

**Table 1:** Patient demographics and baseline characteristics.

Characteristics	Unmatched			Matched		
	Unmarried, N = 16,567^a^	Married, N = 14,286^b^	*p*-value^b^	Unmarried, N = 10,771^a^	Married, N = 10,771^a^	*p*-value^b^
Year of diagnosis			< 0.001			0.838
2000–2008	7864 (47%)	7290 (51%)		5234 (49%)	5249 (49%)	
2009–2017	8703 (53%)	6996 (49%)		5537 (51%)	5522 (51%)	
Age			< 0.001			0.246
< 45 years	6761 (41%)	6697 (47%)		4982 (46%)	5057 (47%)	
45–60 years	5536 (33%)	5350 (37%)		3880 (36%)	3763 (35%)	
> 60 years	4270 (26%)	2239 (16%)		1909 (18%)	1951 (18%)	
Race			< 0.001			0.088
White	12,215 (74%)	11,404 (80%)		8806 (82%)	8887 (83%)	
Black	2941 (18%)	1019 (7%)		889 (8%)	903 (8%)	
Other	1411 (9%)	1863 (13%)		1076 (10%)	981 (9%)	
Ethnicity			< 0.001			0.753
Hispanic	4166 (25%)	3299 (23%)		2690 (25%)	2710 (25%)	
Non-Hisp	12,401 (75%)	10,987 (77%)		8081 (75%)	8061 (75%)	
Median household income			< 0.001			0.404
< 75,000 USD	9433 (57%)	7403 (52%)		5782 (54%)	5843 (54%)	
≥ 75,000 USD	7134 (43%)	6883 (48%)		4989 (46%)	4928 (46%)	
Residence			< 0.001			0.311
Urban	14,743 (89%)	12,481 (87%)		9525 (88%)	9477 (88%)	
Rural	1824 (11%)	1805 (13%)		1246 (12%)	1294 (12%)	
Histology			< 0.001			0.054
SCC	11,887 (72%)	8762 (61%)		7421 (69%)	7266 (67%)	
AC	3068 (19%)	3877 (27%)		2334 (22%)	2409 (22%)	
Others	1612 (10%)	1647 (12%)		1016 (9%)	1096 (10%)	
Grade			< 0.001			0.077
Well	1943 (12%)	2389 (17%)		1430 (13%)	1437 (13%)	
Moderately	7066 (43%)	5989 (42%)		4667 (43%)	4509 (42%)	
Poorly / Undiff	7558 (46%)	5908 (41%)		4674 (43%)	4825 (45%)	
Stage			< 0.001			0.748
Localized	6810 (41%)	7537 (53%)		5251 (49%)	5302 (49%)	
Regional	7209 (44%)	5117 (36%)		4237 (39%)	4184 (39%)	
Distant	2548 (15%)	1632 (11%)		1283 (12%)	1285 (12%)	
Surgery			< 0.001			0.524
Performed	8892 (54%)	9808 (69%)		6821 (63%)	6866 (64%)	
Not performed	7675 (46%)	4478 (31%)		3950 (37%)	3905 (36%)	
Radiotherapy			< 0.001			0.543
Yes	10,608 (64%)	7835 (55%)		6324 (59%)	6280 (58%)	
No	5959 (36%)	6451 (45%)		4447 (41%)	4491 (42%)	
Chemotherapy			< 0.001			0.496
Yes	8758 (53%)	6779 (47%)		5513 (51%)	5463 (51%)	
No/Unknown	7809 (47%)	7507 (53%)		5258 (49%)	5308 (49%)	

^a^n (%).

^b^Pearson’s Chi-squared test.

Before PSM, significant differences were observed between unmarried and married patients in various aspects, including year of diagnosis (*p* < 0.001), age (*p* < 0.001), race (*p* < 0.001), ethnicity (*p* < 0.001), median household income (*p* < 0.001), place of residence (*p* < 0.001), tumor histology (*p* < 0.001), tumor grade (*p* < 0.001), cancer stage (*p* < 0.001), surgery (*p* < 0.001), radiotherapy (*p* < 0.001), and chemotherapy (*p* < 0.001). After 1:1 PSM, 10,771 unmarried patients and 10,771 married patients were included in the analysis, with no significant differences in any of the included variables (all *p* > 0.05).

### Survival outcomes

**Fig. 2:**
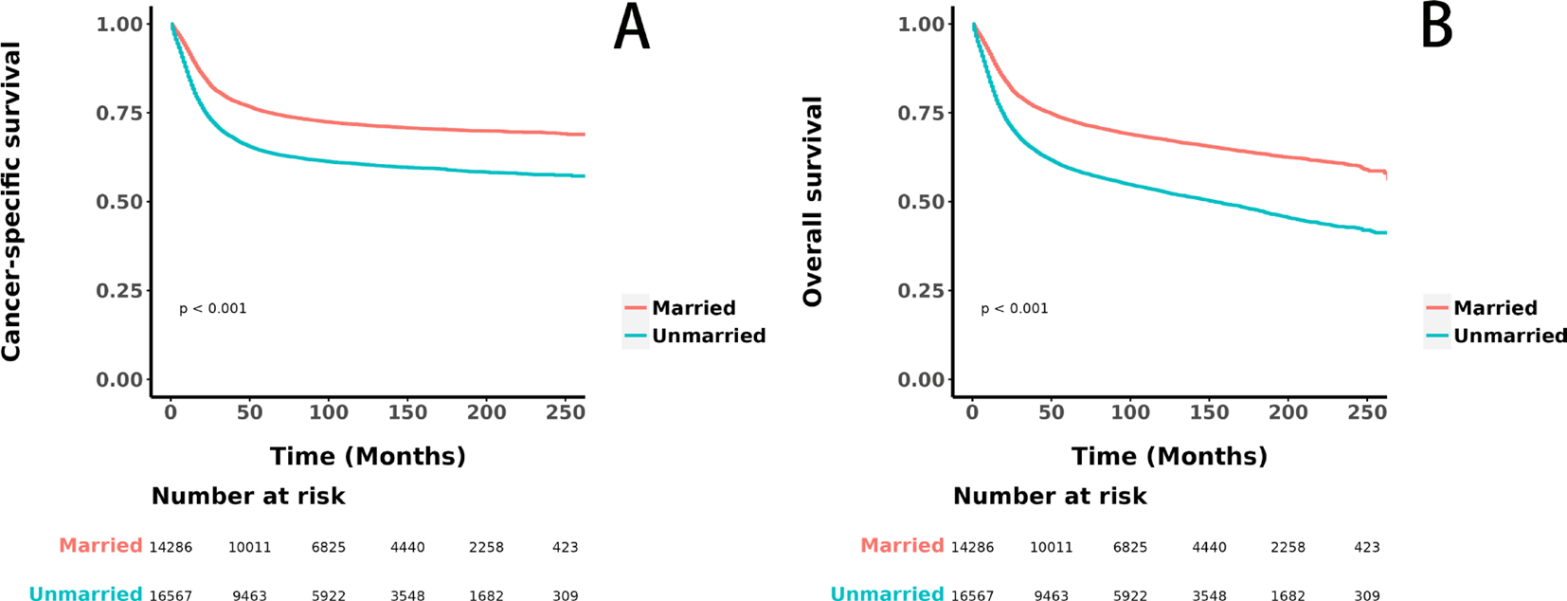
Kaplan–Meier curves illustrating cancer-specific survival (**A**) and overall survival (**B**) before propensity score matching.

**Fig. 3:**
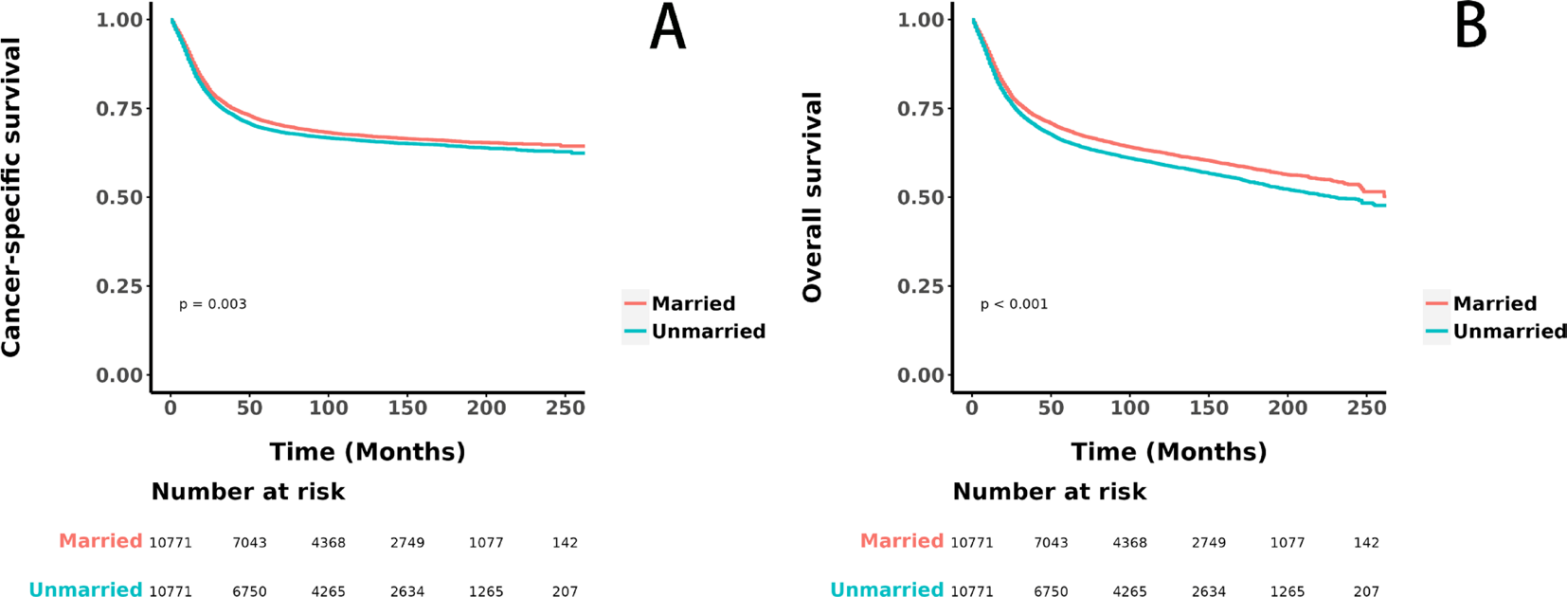
Kaplan–Meier curves illustrating cancer-specific survival (**A**) and overall survival (**B**) after propensity score matching.

Before PSM, unmarried patients exhibited significantly lower CSS (HR = 1.55, 95% CI 1.49–1.61, *p* < 0.001, Fig. 2A) and OS (HR = 1.66, 95% CI 1.61–1.73, *p* < 0.001, Fig. 2B) compared to married patients. After adjustment with 1:1 PSM, although the differences were reduced, unmarried patients still had significantly lower CSS (HR = 1.07, 95% CI 1.02–1.13, *p* = 0.003, see Fig. 3A) and OS (HR = 1.13, 95% CI 1.08–1.18, *p* < 0.001, see Fig. 3B) than married patients. Table 2 details the differences in CSS and OS at key time points of 12 months, 36 months, and 60 months for the two groups before and after PSM. Prior to PSM, unmarried patients had markedly lower CSS at 12 months (84.0% vs. 91.0%), 36 months (69.0% vs. 79.0%), and 60 months (64.0% vs. 75.0%), and similarly lower OS at 12 months (82.0% vs. 91.0%), 36 months (66.0% vs. 78.0%), and 60 months (60.0% vs. 73.0%) compared to married patients. Even after PSM, the CSS at 12 months (88.0% vs. 90.0%), 36 months (74.0% vs. 76.0%), and 60 months (69.0% vs. 71.0%), and the OS at 12 months (87.0% vs. 89.0%), 36 months (71.0% vs. 74.0%), and 60 months (66.0% vs. 69.0%) for unmarried patients remained significantly lower than those for married patients, indicating a less favorable prognosis.

**Table 2:** Differences in cancer-specific survival and overall survival at 12, 36, and 60 months between marital status before and after propensity score matching.

Characteristic	12-Month	36-Month	60-Month	*p*-value^a^
CSS before PSM				
Overall	87% (87%, 88%)	74% (73%, 74%)	69% (69%, 70%)	
Married	91% (91%, 92%)	79% (78%, 80%)	75% (75%, 76%)	< 0.001
Unmarried	84% (83%, 85%)	69% (68%, 69%)	64% (63%, 65%)	
OS before PSM				
Overall	86% (86%, 87%)	71% (71%, 72%)	66% (65%, 66%)	
Married	91% (90%, 91%)	78% (77%, 78%)	73% (72%, 74%)	< 0.001
Unmarried	82% (82%, 83%)	66% (65%, 66%)	60% (59%, 60%)	
CSS after PSM				
Overall	89% (89%, 89%)	75% (74%, 75%)	70% (70%, 71%)	
Married	90% (89%, 91%)	76% (75%, 77%)	71% (70%, 72%)	0.003
Unmarried	88% (87%, 89%)	74% (73%, 75%)	69% (68%, 70%)	
OS after PSM				
Overall	88% (87%, 88%)	73% (72%, 73%)	67% (67%, 68%)	
Married	89% (88%, 90%)	74% (73%, 75%)	69% (68%, 70%)	< 0.001
Unmarried	87% (86%, 87%)	71% (71%, 72%)	66% (65%, 66%)	

CSS, cancer-specific survival; PSM, propensity score matching; OS, overall survival.

^a^Log-rank test.

**Fig. 4:**
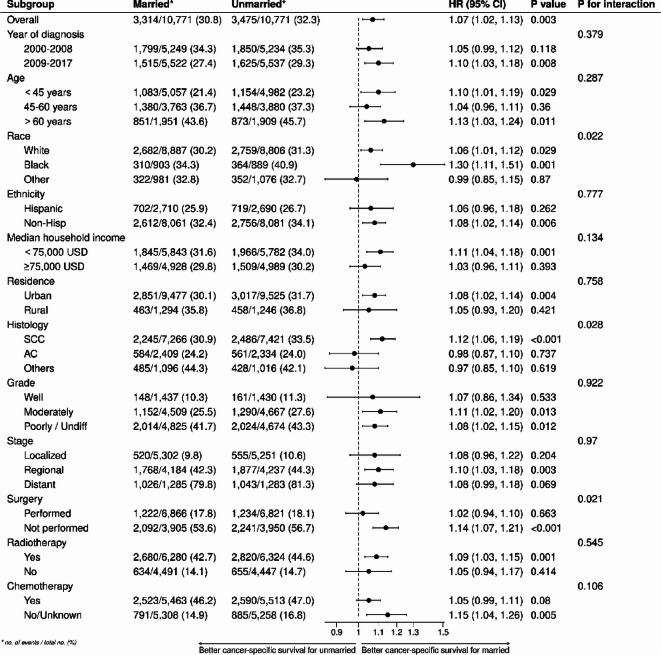
Forest plot of subgroup analysis of cancer-specific survival.

**Fig. 5:**
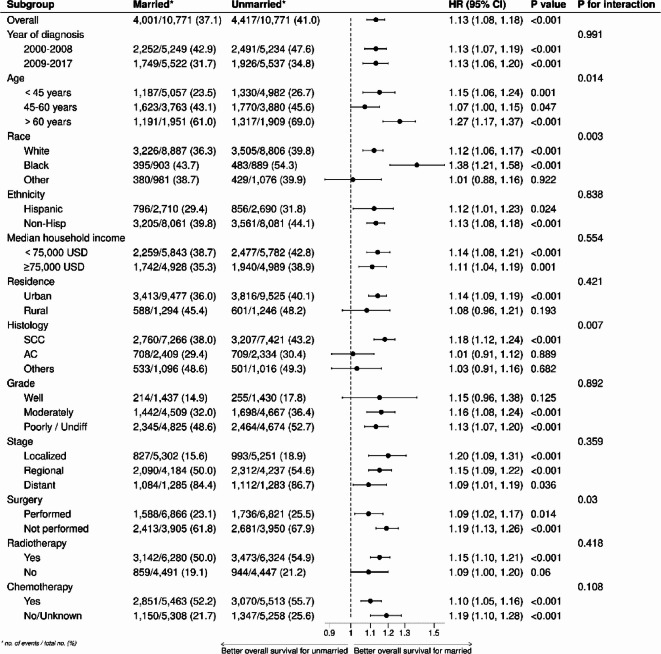
Forest plot of subgroup analysis of overall survival.

In subgroup analyses, as depicted in Fig. 4, compared to married patients, unmarried patients exhibited lower CSS in the majority of subgroups (HR > 1, *p* < 0.05). Furthermore, there were no significant differences in CSS among patients in the following subgroups (diagnosed between 2000 and 2008, aged 45–60 years, of other races, Hispanic ethnicity, with a median household income ≥ 75,000 USD, living in rural areas, with adenocarcinoma and other histological types, well-differentiated tumors, localized and distant metastatic stages, who underwent surgery, did not receive radiotherapy, and received chemotherapy) (all *p* > 0.05). As shown in Fig. 5, except for the subgroups (of other races, living in rural areas, with adenocarcinoma and other histological types, well-differentiated tumors, and not receiving radiotherapy) where no significant impact of marital status on OS was observed (all *p* > 0.05), in the remaining subgroups, the OS of unmarried patients was significantly lower than that of married patients (HR > 1, *p* < 0.05).

In the multivariate Cox regression analysis, the year of diagnosis, age, marital status, race, ethnicity, median household income, histological type, tumor grade, stage, surgery, and chemotherapy were identified as independent prognostic factors for both CSS and OS (all with *p* < 0.05), while radiotherapy was identified as an independent prognostic factor solely for OS (*p* < 0.001). The results of the Cox regression are presented in Table 3.

**Table 3:** Univariate and multivariate Cox regression for cancer-specific survival and overall survival.

Characteristic	Cancer-specific survival				Overall survival			
	Univariate		Multivariate		Univariate		Multivariate	
	HR (95% CI)	*p*-value	HR (95% CI)	*p*-value	HR (95% CI)	*p*-value	HR (95% CI)	*p*-value
Year of diagnosis								
2000–2008	Ref		Ref		Ref		Ref	
2009–2017	0.88 (0.84, 0.92)	< 0.001	0.86 (0.82, 0.90)	< 0.001	0.88 (0.84, 0.92)	< 0.001	0.87 (0.83, 0.91)	< 0.001
Age								
< 45 years	Ref		Ref		Ref		Ref	
45–60 years	1.87 (1.77, 1.97)	< 0.001	1.18 (1.11, 1.25)	< 0.001	2.03 (1.93, 2.14)	< 0.001	1.37 (1.30, 1.45)	< 0.001
> 60 years	2.65 (2.49, 2.82)	< 0.001	1.46 (1.36, 1.56)	< 0.001	3.60 (3.40, 3.80)	< 0.001	2.14 (2.02, 2.27)	< 0.001
Marital status								
Married	Ref		Ref		Ref		Ref	
Unmarried	**1.07 (1.02, 1.13)**	**0.003**	**1.12 (1.07, 1.17)**	**< 0.001**	**1.13 (1.08, 1.18)**	**< 0.001**	**1.19 (1.14, 1.24)**	**< 0.001**
Race								
White	Ref		Ref		Ref		Ref	
Black	1.28 (1.19, 1.39)	< 0.001	1.14 (1.05, 1.24)	0.001	1.34 (1.25, 1.44)	< 0.001	1.17 (1.09, 1.26)	< 0.001
Other	1.10 (1.02, 1.19)	0.019	0.90 (0.83, 0.98)	0.013	1.08 (1.00, 1.16)	0.037	0.84 (0.78, 0.91)	< 0.001
Ethnicity								
Hispanic	Ref		Ref		Ref		Ref	
Non-Hisp	1.24 (1.17, 1.31)	< 0.001	1.17 (1.10, 1.24)	< 0.001	1.34 (1.27, 1.41)	< 0.001	1.24 (1.17, 1.31)	< 0.001
Median household income								
< 75,000USD	Ref		Ref		Ref		Ref	
≥ 75,000USD	0.89 (0.85, 0.94)	< 0.001	0.94 (0.90, 0.99)	0.026	0.88 (0.85, 0.92)	< 0.001	0.93 (0.89, 0.97)	0.001
Residence								
Urban	Ref		Ref		Ref		Ref	
Rural	1.22 (1.14, 1.31)	< 0.001	1.01 (0.94, 1.09)	0.719	1.29 (1.21, 1.37)	< 0.001	1.06 (0.99, 1.13)	0.114
Histology								
SCC	Ref		Ref		Ref		Ref	
AC	0.71 (0.66, 0.75)	< 0.001	1.20 (1.12, 1.28)	< 0.001	0.69 (0.66, 0.74)	< 0.001	1.12 (1.05, 1.19)	< 0.001
Others	1.49 (1.39, 1.60)	< 0.001	1.59 (1.47, 1.71)	< 0.001	1.35 (1.26, 1.44)	< 0.001	1.48 (1.38, 1.58)	< 0.001
Grade								
Well	Ref		Ref		Ref		Ref	
Moderately	2.71 (2.41, 3.06)	< 0.001	1.61 (1.43, 1.82)	< 0.001	2.31 (2.09, 2.54)	< 0.001	1.45 (1.32, 1.61)	< 0.001
Poorly/Undiff	5.02 (4.47, 5.63)	< 0.001	2.22 (1.97, 2.51)	< 0.001	3.98 (3.62, 4.38)	< 0.001	1.96 (1.77, 2.16)	< 0.001
Stage								
Localized	Ref		Ref		Ref		Ref	
Regional	5.45 (5.09, 5.84)	< 0.001	3.57 (3.27, 3.89)	< 0.001	4.01 (3.79, 4.23)	< 0.001	2.54 (2.37, 2.72)	< 0.001
Distant	20.39 (18.92, 21.97)	< 0.001	11.53 (10.50, 12.66)	< 0.001	13.90 (13.04, 14.81)	< 0.001	7.64 (7.06, 8.27)	< 0.001
Surgery								
Performed	Ref		Ref		Ref		Ref	
Not performed	4.59 (4.36, 4.82)	< 0.001	2.05 (1.93, 2.17)	< 0.001	4.16 (3.98, 4.35)	< 0.001	2.04 (1.93, 2.16)	< 0.001
Radiotherapy								
Yes	Ref		Ref		Ref		Ref	
No	0.27 (0.26, 0.29)	< 0.001	0.93 (0.85, 1.00)	0.062	0.31 (0.29, 0.32)	< 0.001	0.87 (0.81, 0.93)	< 0.001
Chemotherapy								
Yes	Ref		Ref		Ref		Ref	
No/Unknown	0.27 (0.26, 0.29)	< 0.001	1.19 (1.11, 1.29)	< 0.001	0.34 (0.32, 0.36)	< 0.001	1.28 (1.20, 1.37)	< 0.001

HR, hazard ratio; CI, confidence interval.

Bold indicates marital status is an independent prognostic factor for cancer-specific survival and overall survival.

### Interaction and sensitivity analyses

None of the interactions reached statistical significance (all *P* > 0.10), and thus the final model was retained without these terms. The detailed results are presented in Supplementary Tables S1 and S2.

Across all sensitivity analyses, unmarried status remained an independent risk factor for both CSS and OS, with hazard-ratio directions consistent with our primary results (Supplementary Table S3).

## Discussion

Over the past three decades, marital status has been proven to be associated with the prognosis of various types of cancer^24^. For instance, María Elena Martínez and her colleagues analyzed data from 145,564 patients with invasive breast cancer from the California Cancer Registry (CCR), finding that in a multivariable-adjusted model, the relative risk of total mortality for unmarried women compared to married women was 1.28 (95% CI 1.24–1.32)^28^. Similarly, Dechang Zhao and others used data from 58,424 patients with non-small cell lung cancer (NSCLC) in the SEER database, discovering that married patients had significantly better OS and CSS compared to unmarried patients, and multivariate COX regression analysis also indicated that being unmarried is an independent risk factor for the prognosis of NSCLC^31^. The above conclusions have also been confirmed in other types of tumors, including glioblastoma in the nervous system^30^, prostate cancer in the urinary system^25,26^, early liver cancer in the digestive system^27^, and pancreatic ductal carcinoma^29^. In the field of gynecological malignant tumors, similar findings have been reported. In 2013, a study by Haider Mahdi and others included 49,777 patients with epithelial ovarian cancer, showing that the 5-year overall survival rate for married patients was 45.0%, while for unmarried patients it was 33.1% (*p* < 0.001). After controlling for confounding factors, married patients had a significantly improved survival rate compared to unmarried patients (HR = 0.8, 95% CI 0.78–0.83, *p* < 0.001)^35^. In 2019, a study by Pei Luo and others included 19,276 patients with serous ovarian cancer, finding that the median overall survival for unmarried and married patients was 48 months and 52 months, respectively, and multivariate Cox regression analysis indicated that the risk ratio (HR) for unmarried patients was 1.05 (95% CI 1.00–1.11; *P* = 0.05)^36^. In the same year, a study by Jia Dong and others included 39,387 patients with endometrial cancer, identifying marital status as an independent prognostic factor for OS and CSS, with married patients having the lowest risk of death^37^.

Current research indicates that the impact of marital status on the prognosis of malignant tumors may be related to several factors. In terms of psychosocial factors, cancer patients endure significant psychological stress, and the emotional support provided by a spouse in specific ways can help reduce the negative impact of stress, thereby leading to better therapeutic outcomes^28^. Studies have also found that individuals who are widowed, divorced, or separated are more likely to suffer from psychological distress during the disease diagnosis and treatment process due to the lack of a partner’s support, increasing the risk of mental health issues^38^. A healthy marital status not only helps maintain a positive psychological state and reduce negative emotions such as anxiety and depression but also plays a crucial role in improving survival rates^39,40^. With the emotional support and encouragement of their spouses, married patients are often better able to cope with stress and depression and are more motivated to seek treatment^41,42^. In addition, the association between optimistic mood and a lower risk of death has been confirmed by prospective studies^43^. In terms of physiological factors, some studies have suggested that marital status may improve patients’ physiological condition by affecting cardiovascular, endocrine, and immune functions, with the quality of marriage playing a decisive role^44,45^. A loving and caring partner is associated with an increased release of oxytocin, a hormone that can inhibit the growth of cancer cells through both indirect and direct mechanisms^46^. In contrast, unmarried patients may be chronically exposed to higher levels of glucocorticoids and catecholamines, which may lead to physiological dysfunction and negatively affect the tumor microenvironment and tumor growth, migration, and angiogenesis, thus impacting prognosis^47^. At the same time, psychological factors have a strong impact on the tumor immune microenvironment^48^; depression and stress can reduce the activity of cytotoxic T cells and natural killer cells, potentially weakening immune surveillance and promoting tumor growth^49^. Research has shown that widowed individuals have a poorer peripheral blood lymphocyte response and reduced activity of natural killer cells, which play a key role in identifying and eliminating cancer cells^24^. HPV infection is a clear cause of cervical cancer, which is a unique aspect of cervical cancer compared to other cancers. Previous studies have indicated that married women have a lower HPV infection rate, especially for high-risk oncogenic HPV types, compared to other unmarried women or women living with partners^50^. In terms of economic factors, a stable marriage is usually associated with a higher economic status, and family members such as spouses and children may provide financial and emotional support for patients’ long-term treatment^51^. Married individuals with a better economic foundation are more likely to have health insurance and receive medical assistance at the time of diagnosis^52^. In terms of compliance, married individuals often adopt healthier lifestyles, including better dietary habits, more exercise, and less drug abuse, which helps improve health outcomes, and a stable marriage can enhance patients’ adherence to treatment regimens^53^. Marriage and cohabitation with others can moderately improve compliance, and patients from cohesive families show higher compliance than those from conflict-ridden families^54^, and higher treatment compliance is closely related to better survival outcomes^55,56^. In addition, married patients are more likely to detect the disease at an early stage and choose active treatment. Compared with unmarried patients, they are more likely to receive chemotherapy and have a higher survival rate, which may be related to the involvement and support of family members in the treatment plan, as well as a higher perceived value of life^25^.

Some previous studies have preliminarily explored the impact of marital status on the prognosis of cervical cancer^32–34^. In 2010, Mehul K Patel and colleagues analyzed 7997 patients with primary invasive cervical cancer from the SEER database and found that the risk of death for unmarried, separated/divorced, and widowed women was 1.13 (95% CI = 1.03–1.25), 1.41 (95% CI = 1.28–1.57), and 2.51 (95% CI = 2.29–2.76) times higher than that for married women, respectively. However, after adjusting for covariates in the model, no independent association was found between marital status and the risk of death (*p* = 0.21)^32^. In 2016, Sanae El Ibrahimi and colleagues studied 31,425 cervical cancer patients from the SEER database, and the results showed that after controlling for relevant confounding factors, the risk of death for unmarried, separated/divorced, and widowed women was significantly increased compared to married women, with hazard ratios of 1.35 (95% CI 1.28–1.43), 1.22 (95% CI 1.15–1.29), and 1.28 (95% CI 1.19–1.36), respectively^33^. Similarly, Qing Chen and colleagues reported using SEER data that married cervical cancer patients had better OS and CSS than unmarried patients, and marital status was an independent prognostic factor for OS (HR: 0.830, 95% CI 0.798–0.862) and CSS (HR: 0.892, 95% CI 0.850–0.937) in cervical cancer patients^34^. Although these studies offered valuable preliminary insights and some had sample sizes comparable to ours, their findings have been inconsistent. Therefore, further large-population investigations into the impact of marital status on CSS and OS remain highly valuable. A systematic and comprehensive study of the impact of marital status on the prognosis of cervical cancer can provide key guidance for personalized treatment, reveal potential biological mechanisms, and help formulate targeted public health policies. Our study used patient data from the SEER database to construct a large retrospective cohort and found that unmarried cervical cancer patients had poorer prognosis, and marital status was an independent prognostic factor for cervical cancer. After adjusting for baseline differences with PSM and controlling for covariates with multivariate COX regression analysis, this difference remained significant. It is worth noting that in all subgroup analyses, the prognosis of unmarried cervical cancer patients was not better than that of married patients. In the subgroup analysis based on histological grading, marital status had a significant impact on the CSS and OS of patients with moderately differentiated and poor/undifferentiated cervical cancer, while the impact on well-differentiated cervical cancer patients was not significant for CSS (*p* = 0.533) and OS (*p* = 0.125). This phenomenon of non-significance may be due to a small sample size, limiting statistical power, especially when detecting smaller differences, a larger sample size is needed to provide sufficient statistical power, which may also be one of the reasons for the non-significance of the results in several other subgroup analyses.

This study has several notable strengths. First and foremost, by leveraging one of the world’s largest cancer databases—the SEER database—this study was able to utilize its extensive patient data resources. This not only enhanced the statistical power of our analysis but also improved the broad representativeness of the research findings. Second, by employing advanced statistical methods such as PSM and multivariate COX regression, we effectively minimized the potential impact of baseline characteristic differences and covariates on the study results, thereby enhancing the credibility of the conclusions. Third, compared to similar studies, this study controlled for a more comprehensive range of potential confounding factors while including a large sample size, which played a key role in increasing the precision of the research findings. However, this study also faces several limitations. Firstly, as a retrospective study, it may be more susceptible to biases than prospective studies. Secondly, due to data limitations in the SEER database, we were unable to obtain all variables that could potentially affect prognosis, such as smoking history, human papillomavirus infection history, sexual behavior history, reproductive history, chronic disease history, comorbidities, specific chemotherapy regimens and dosages, and special pathological features like lymphovascular space invasion, parametrial invasion, and vaginal margin status, all of which could confound the study results. Thirdly, the SEER database only records the marital status of patients at the time of disease diagnosis and does not update it during the follow-up process, which may introduce certain information biases. Additionally, the SEER database does not record the quality of patients’ marriages, limiting our in-depth exploration of how marital quality affects the prognosis of cervical cancer patients. Existing studies have shown that the closeness of marital relationships has an important impact on the psychological adaptation of cancer patients^57^. Poor marital relationships may lead to higher levels of dysfunction and worse health behaviors, thereby affecting the prognosis of cancer^58^. Fourthly, the SEER database only records legally recognized marriages and does not document de facto cohabiting partner relationships; our study classified cohabiting partners as unmarried, yet patients with cohabiting partners may receive equal emotional and socioeconomic support. Although the proportion of such patients is small, it may still affect the study results. Fifthly, the absence of recurrence and quality-of-life (QoL) information in SEER prevented us from assessing disease-free survival (DFS) and QoL, key indicators of long-term prognosis. Future prospective studies should incorporate these endpoints to provide a more comprehensive assessment. Lastly, since the SEER database mainly covers white and black populations, this may limit the applicability of our research findings to other racial groups, such as individuals of Asian descent. Therefore, well-designed, large-scale prospective studies that include a more diverse range of racial groups are needed in the future to further verify the impact of marital status on the prognosis of cervical cancer.

## Conclusion

In summary, our study, based on an in-depth analysis of a large sample of data, has revealed a significant association between unmarried status and poor prognosis in cervical cancer, confirming marital status as an independent prognostic indicator. However, to further validate these findings and gain a deeper understanding of the underlying biological mechanisms, we urgently need to conduct more prospective studies and mechanistic investigations. At the same time, it is necessary to carry out further research to explore the impact of marital satisfaction and quality on the prognosis of cervical cancer patients, with the aim of improving their outcomes.

## Methods

### Data source

The SEER database is a comprehensive cancer statistics database managed by the National Cancer Institute. It collects data on the incidence, prevalence, survival, and mortality rates of cancer in each region of the United States. This study used the SEER-17 dataset from 2000 to 2021, covering approximately 26.5% of the total U.S. population.

### Patient selection

In this study, we identified primary cervical cancer cases from 2000 to 2021 in the SEER-17 data set using the codes C53.0, C53.1, C53.8, and C53.9, based on the International Classification of Diseases in Oncology, Third Edition (ICD-O-3) coding system. The study was limited to adults aged 18 years and older, all of whom were histologically confirmed and each patient was assured of having only one primary malignant tumor, cervical cancer. During the screening process, we excluded cases with zero or unknown follow-up time and those with unclear information on oncology outcomes, including cancer-specific survival and overall survival. In addition, patients whose marital status was unknown at diagnosis, as well as cases with missing information in other subgroups (race, ethnicity, median household income, place of residence, histological type, grade, stage, surgery, radiotherapy, chemotherapy), were not included in the final analysis. In the end, 30,853 patients were enrolled in the study.

### Variable processing

Based on the coding system of the SEER database, we obtained patients’ demographic characteristics (including age, year of diagnosis, marital status, race, ethnicity, median household income, and place of residence), oncology characteristics (including histological type, grade, stage, and regional lymph nodes), and treatment measures (surgery, radiation, and chemotherapy). In addition, we determined tumor prognosis, including cancer-specific survival (CSS) and overall survival (OS), based on the patient’s final state and duration of follow-up. In the SEER-17 dataset from 2000 to 2021, cancer staging is classified into three categories: “Localized” who is limited to the primary organ, “Regional” who has spread to nearby tissues, and “Distant” who has metastasized far away. Median household income (adjusted to 2022) was categorized as follows: < 40,000 United States Dollar (USD), 40,000–44,999 USD, 45,000–49,999 USD, 50,000–54,999 USD, 55,000–59,999 USD, 60,000–64,999 USD, 65,000–69,999 USD, 70,000–74,999 USD, 75,000–79,999 USD, 80,000–84,999 USD, 85,000–89,999 USD, 90,000–94,999 USD, 95,000–99,999 USD, 100,000–109,999 USD, 110,000–119,999 USD, and 120,000 + USD. Marital status was classified as Married, Single (never married), Separated, Divorced, Widowed, and Unmarried or Domestic Partner.

In the subsequent statistical analysis, we divided the years of diagnosis into 2000–2008 and 2009–2017, the age of patients at diagnosis into < 45 years, 40–60 years, > 60 years, and the marital status at diagnosis into married and unmarried (including single, separated, divorced, widowed, and unmarried or domestic partner). Racial groups were classified as white, black, and other (Asian or Pacific Islander and American Indian/Native Alaska), ethnic groups as Hispanic and non-Hispanic, median household income as < 75,000 USD and ≥ 75,000 USD, and residence as urban and rural. These cases were classified into squamous cell carcinoma, adenocarcinoma and other types based on histological coding. Histological grades were Well, Moderately and Poorly/Undifferentiated.

### Statistical analysis

The patients were divided into married and unmarried groups according to their marital status. Differences in baseline features between the two groups were determined by Wilcoxon rank sum test and Pearson Chi-square test, as appropriate. The tumor outcome measures we were interested in were OS and CSS. Kaplan–Meier curves and Log-rank tests were used to assess differences in CSS and OS between the two groups. Subsequently, to reduce the impact of baseline differences on survival, we performed 1:1 propensity score matching (PSM). After PSM, Kaplan–Meier curves and Log-rank tests were used again to assess the effects of marital status on CSS and OS. In addition, subgroup analyses were performed based on predetermined variables. Finally, multivariate COX regression analysis was used to verify the independent effects of marital status on CSS and OS. In univariate COX regression, variables with a *p* value less than 0.05 were included in multivariate COX regression. To assess whether marital status interacts with socioeconomic characteristics (race, ethnicity, median household income, and residence), we included interaction terms in the multivariable Cox models and evaluated their significance using likelihood-ratio tests (LRTs). To assess the robustness of our findings across different matching methods and model assumptions, we conducted three sensitivity analyses (stabilized inverse-probability-of-treatment weighting as an alternative to PSM; full-cohort multivariable regression to adjust for confounding; re-classifying cohabiting but unmarried partners as “married” to evaluate the impact of cohabitation). All statistical analyses in this study were performed using R software (version 4.3.0). All P-values were bilateral, and the threshold of significance was set at 0.05.

## Supplementary Information

Supplementary Information 1.

Supplementary Information 2.

Supplementary Information 3.

## Data Availability

The data that support the findings of this study are openly available in software package SEER*Stat 8.4.3 (https://seer.cancer.gov/seerstat/).
