# Supplementary material for: The effect of marital status on cervical cancer related prognosis: a propensity score matching study
Source: Sci Rep. 2025 Oct 8;15:35166. doi: 10.1038/s41598-025-19122-3 (PMC12508153; doi:10.1038/s41598-025-19122-3)
Supplement: Supplementary file 1 — Supplementary Information 1. [file 41598_2025_19122_MOESM1_ESM.docx]

Supplementary Table S1: Interaction Effects Between Marital Status and Selected Covariates on Cancer-Specific Survival.

| **Characteristic** | **N** | **Event N** | **HR** | **95% CI** | **p-value** |
| --- | --- | --- | --- | --- | --- |
| **Marital status * Race** | 30,853 | 10,060 |  |  |  |
| Unmarried * Black | 2,941 | 1,318 | 1.03 | 0.90, 1.16 | 0.686 |
| Unmarried * Other | 1,411 | 512 | 1.02 | 0.90, 1.17 | 0.740 |
| **Marital status * Ethnicity** | 30,853 | 10,060 |  |  |  |
| Unmarried * Non-Hisp | 12,401 | 4,895 | 1.07 | 0.97, 1.19 | 0.164 |
| **Marital status * Median household income** | 30,853 | 10,060 |  |  |  |
| Unmarried * ≥75,000 USD | 7,134 | 2,508 | 0.96 | 0.89, 1.04 | 0.324 |
| **Marital status * Residence** | 30,853 | 10,060 |  |  |  |
| Unmarried * Rural | 1,824 | 739 | 1.06 | 0.94, 1.20 | 0.309 |
| Abbreviations: CI = Confidence Interval, HR = Hazard Ratio | | | | | |
