# Supplementary material for: The effect of marital status on cervical cancer related prognosis: a propensity score matching study
Source: Sci Rep. 2025 Oct 8;15:35166. doi: 10.1038/s41598-025-19122-3 (PMC12508153; doi:10.1038/s41598-025-19122-3)
Supplement: Supplementary file 2 — Supplementary Information 2. [file 41598_2025_19122_MOESM2_ESM.docx]

Supplementary Table S2: Interaction Effects Between Marital Status and Selected Covariates on Overall Survival.

| **Characteristic** | **N** | **Event N** | **HR** | **95% CI** | **p-value** |
| --- | --- | --- | --- | --- | --- |
| **Marital status * Race** | 30,853 | 12,545 |  |  |  |
| Unmarried * Black | 2,941 | 1,711 | 1.00 | 0.89, 1.11 | 0.942 |
| Unmarried * Other | 1,411 | 624 | 1.00 | 0.89, 1.13 | 0.972 |
| **Marital status * Ethnicity** | 30,853 | 12,545 |  |  |  |
| Unmarried * Non-Hisp | 12,401 | 6,296 | 1.07 | 0.97, 1.17 | 0.157 |
| **Marital status * Median household income** | 30,853 | 12,545 |  |  |  |
| Unmarried * ≥75,000 USD | 7,134 | 3,216 | 1.02 | 0.95, 1.10 | 0.565 |
| **Marital status * Residence** | 30,853 | 12,545 |  |  |  |
| Unmarried * Rural | 1,824 | 983 | 1.03 | 0.92, 1.14 | 0.636 |
| Abbreviations: CI = Confidence Interval, HR = Hazard Ratio | | | | | |
