# Supplementary material for: The effect of marital status on cervical cancer related prognosis: a propensity score matching study
Source: Sci Rep. 2025 Oct 8;15:35166. doi: 10.1038/s41598-025-19122-3 (PMC12508153; doi:10.1038/s41598-025-19122-3)
Supplement: Supplementary file 3 — Supplementary Information 3. [file 41598_2025_19122_MOESM3_ESM.docx]

**Supplementary Table S3: Related Sensitivity Analyses and Results.**

| **Different matching methods or model assumptions** | **Marital status** | **Cancer-specific survival** | | **Overall survival** | |
| --- | --- | --- | --- | --- | --- |
|  |  | **HR (95% CI)** | **p-value** | **HR (95% CI)** | **p-value** |
| **1:1 PSM (Used in the manuscript)** | Married | Ref |  | Ref |  |
|  | Unmarried | 1.07 (1.02-1.13) | 0.003 | 1.13 (1.08, 1.18) | <0.001 |
|  |  |  |  |  |  |
| **Inverse Probability of Treatment Weighting** | Married | Ref |  | Ref |  |
|  | Unmarried | 1.16 (1.11, 1.21) | <0.001 | 1.11 (1.07, 1.16) | <0.001 |
|  |  |  |  |  |  |
| **Full-cohort Multivariable regression** | Married | Ref |  | Ref |  |
|  | Unmarried | 1.20 (1.15, 1.25) | <0.001 | 1.27 (1.22, 1.31) | <0.001 |
|  |  |  |  |  |  |
| **Classify "Domestic Partner" as "Married"** | Married | Ref |  | Ref |  |
|  | Unmarried | 1.13 (1.08, 1.19) | <0.001 | 1.20 (1.15, 1.25) | <0.001 |

Abbreviation: PSM, Propensity Score Matching; HR, Hazard Ratio; CI, Confidence Interval.
